# Editorial: The metabolic pathways of archaea

**DOI:** 10.3389/fmicb.2025.1648560

**Published:** 2025-07-08

**Authors:** Hannu Myllykallio, Wei Qin, Ivan A. Berg

**Affiliations:** ^1^LOB, CNRS, INSERM, École Polytechnique, Institut Polytechnique de Paris, Palaiseau, France; ^2^School of Biological Sciences and Institute for Environmental Genomics, The University of Oklahoma, Norman, OK, United States; ^3^Institute for Molecular Microbiology and Biotechnology, University of Münster, Münster, Germany

**Keywords:** archaea, thermoacidophiles, ammonia-oxidizing archaea, genome annotation, mevalonate pathway

Archaea are fascinating microorganisms that have been recognized as the third domain of life for the past 40 years. Archaea are ubiquitous, inhabiting soil and marine environments, marshlands, engineered systems, and the gastrointestinal tracts of animals, along with some of the most hostile environments on Earth. Although archaea play a crucial role in the global biogeochemical cycle, our understanding of their metabolic diversity is still limited. Research into the metabolic pathways of archaea expands our understanding of the evolution and origins of life and their adaptive and biotechnological potential in diverse environments. The goal of the Research Topic “The Metabolic Pathways of Archaea” was therefore to improve our understanding of their metabolic processes.

Blake et al. studied the growth of the thermoacidophilic archaeon *Metallosphaera sedula* under mixotrophic and heterotrophic conditions. This organism can use a variety of organic and inorganic electron donors to perform oxidative phosphorylation. The authors found that the mixotrophic cells cultured on yeast extract in the presence of soluble iron bifurcated into coccoidal cells of two sizes: smaller cells with an average diameter of 0.6 μm, and larger cells with an average diameter of 1.35 μm. The authors discuss the physiological reasons for this phenotypic heterogeneity.

The metabolomes of the iron- and sulfur-oxidizing thermoacidophilic archaea remain poorly characterized, as the isolation of organic molecules from these microorganisms is challenging. Gfellner et al. developed an improved protocol for their extraction by breaking the tight contacts between cells and the mineral surface, thus facilitating metabolomics analyses of lipophilic compounds using mass spectrometry. This approach led to the identification of molecules implicated in microbial and cell surface interactions through biofilm formation and cell–cell interactions. Moreover, it led to the identification of saturated thiophene-bearing quinones as potential biomarkers for detecting microbial life in extreme environmental conditions in the presence of mineral pyrite (FeS_2_).

Karavaeva and Sousa emphasized the importance of curated genomic annotations for understanding the physiology and metabolism of the many recently discovered archaeal lineages at the phylum level. The authors found that despite the extensive use of the different bioinformatics approaches, major portions of archaeal genomes remain unexplored and poorly understood, thus confirming a substantial gap between automatic classification tools and our understanding of archaeal metabolism. This study indicates the need for further functional studies of archaeal genomes by developing more precise computational methods.

Hernández-Magaña and Kraft investigated abundant ammonia-oxidizing archaea (AOA) as a key player in marine nitrogen cycles. They focused particularly on their potential contribution to nitrogen transformation in hypoxic and oxygen-depleted waters. This is of particular interest as AOA produce significant amounts of the potent greenhouse gas nitrous oxide (N_2_O) as a byproduct of ammonia oxidation. Using the isotope labeling and oxygen-depleted incubations, the authors tracked the origin and fate of the nitrogen gases N_2_O and N_2_ during nitric oxide (NO) dismutation. The presented results provide solid evidence for the role of AOA isolates in this reaction. This article builds upon the authors' previous work on a single AOA model species and demonstrates that oxygen production via NO dismutation to support archaeal ammonia oxidation may be widespread across multiple AOA species and lineages, thereby paving the way for future studies to understand the global environmental role of the sources and sinks of N_2_O in marine oxygen minimum zones.

Liu et al., collected and analyzed 70 complete genomes to analyze the ability of AOA and bacteria (AOB) to use labile-dissolved organic nitrogen (LDON) compounds as a source of nitrogen. Genomic analyses revealed that urea and cyanates are commonly used by these groups of microbes to satisfy their nitrogen demands, thus potentially influencing the choice of their ecological niches. The authors also performed phylogenetic and bioinformatics analyses that indicated the importance of the lateral gene transfer of the relevant metabolic pathways between bacteria and archaea. The reported results thus indicate that LDON utilization is a common feature among AOA and AOB.

The mevalonate pathway is an essential metabolic pathway that produces two five-carbon building blocks called isopentenyl pyrophosphate (IPP) and dimethylallyl pyrophosphate (DMAPP), which are required for the biosynthesis of a large number of isoprenoids. In archaea, this pathway uses a specific intermediate, *trans*-anhydromevalonate phosphate. The synthesis of this unique intermediate is catalyzed by an archaea-specific phosphomevalonate dehydratase that belongs to the aconitase X family of the aconitase superfamily. Komeyama et al., investigated the poorly understood enzymatic mechanism of the archaeal phosphomevalonate dehydratase by reconstructing the iron–sulfur cluster of phosphomevalonate dehydratase from the hyperthermophilic archaeon *Aeropyrum pernix*. The authors report the biochemical and enzymatic characterization of this enzyme. Detailed biophysical and site-directed mutagenesis studies revealed that three conserved cysteine residues coordinate, surprisingly, a [4Fe-4S] cluster, in contrast to bacterial aconitase X family enzymes harboring a [2Fe-2S] cluster.

Microbial ammonia oxidation is a key and potentially rate-limiting step in the global nitrogen cycle. Melcher et al., reported a number of studies addressing biomass productivity and the physiological response of the model soil AOA species *Nitrososphaera viennensis* to different ammonia and carbon dioxide (CO_2_) concentrations under both closed batch and continuous culture conditions. This study revealed quantitative data on the physiology of *N. viennensis* that are important for understanding how biomass production is controlled by AOA. This study also demonstrates that bioprocess technology can be used to understand environmental factors influencing the regulation of archaeal physiology, particularly the interplay between ammonia oxidation and carbon fixation. In addition, since traditional batch cultivation of AOA often yields low biomass, the use of continuous cultivation in chemostats enabled high-biomass production, making previously challenging investigations feasible—such as biofilm composition analysis, purification of key metabolic enzymes, and the subsequent biochemical characterization.

